# Efficacy of ICIs on patients with oncogene-driven non-small cell lung cancer: a retrospective study

**DOI:** 10.20517/cdr.2021.85

**Published:** 2022-01-04

**Authors:** Xiaojin Guo, He Du, Jiayu Li, Menghang Yang, Anweng Xiong, Haiping Zhang, Fengying Wu

**Affiliations:** ^1^Department of Immuno-oncology, The Fourth Hospital of Hebei Medical University, Shijiazhuang 050011, Hebei, China.; ^2^Department of Medical Oncology, Shanghai Pulmonary Hospital, School of Medicine, Tongji University, Shanghai 200000, China.

**Keywords:** Non-squamous NSCLC, driver mutations, immune checkpoint inhibitor

## Abstract

**Aim:** The objective of our study was to assess the efficacy of immune checkpoint inhibitors (ICIs) on patients with non-small-cell lung cancer (NSCLC) harboring oncogenic alterations.

**Methods:** We retrospectively enrolled patients with advanced non-squamous NSCLC who were treated with anti-PD-1-based monotherapy or combined immunotherapy. Major characteristics including PD-L1 expression, treatment, and survival were analyzed.

**Results: **In total, 309 non-squamous NSCLC patients with a median age of 61 years (range 20-88 years) including 70.9% male were retrospectively enrolled. The molecular alterations involved epidermal growth factor receptor (EGFR) (*n* = 81), V-Ki-ras2 Kirsten rat sarcoma viral oncogene homolog (KRAS) (*n* = 31), anaplastic lymphoma kinase (ALK) (*n* = 1), human epidermal growth factor receptor 2 (HER2) (*n* = 12), V-raf murine sarcoma viral oncogene homolog (BRAF) (*n* = 2), rearranged during transfection (*n* = 4), and c-ros oncogene 1 (ROS1) (*n* = 3). In the EGFR subset, the ORR was 30.9% (*n* = 81) and PFS was significantly shorter than WT group (median PFS: 5.7 months *vs. *7.1 months; *P* = 0.0061). In subgroup analyses, ICI combined therapy was significantly correlated with a longer PFS compared with ICI monotherapy (median PFS: 7.7 months *vs. *4.7 months; *P* = 0.0112). In KRAS patients, ORR was 51.6% (*n* = 31). No significant difference was found in subgroup analyses. The ORR and PFS were 16.7% (*n* = 12) and 28.6% (*n* = 7), 7.8 months and 9.0 months for HER2 and EGFR Exon20 insertion patients, respectively. Three ROS1 patients were enrolled with a PFS of 16.0, 34.2, and 45.0 months individually, and one ALK patient with PFS of 4.4 months was identified. No response was found in two BRAF patients.

**Conclusion: **ICI-based combination therapy can bring benefit to patients with EGFR-mutant NSCLC. ICI-based combination therapy could be considered for patients with ROS1 rearrangement, HER2 mutation and EGFR Exon20 insertion NSCLC.

## INTRODUCTION

Lung cancer is the most common cancer worldwide, in terms of both incidence and mortality^[1]^. Over the past decades, great advancements have been achieved, which are attributed to the understanding of tumor biology and the molecular mechanism of tumor progression. The use of small molecule tyrosine kinase inhibitors (TKIs) has dramatically improved the prognosis of patients with specific genomic aberrations^[2-5]^. However, despite the high response of TKIs, acquired resistance inevitably occurs and limits the long-term benefits^[6]^. Once this happens, the subsequent anti-tumor treatment is limited. 

Immune checkpoint inhibitors (ICIs), specifically those targeting PD-1 or programmed death-ligand 1 (PD-L1), have rapidly transformed the treatment paradigm for non-small cell lung cancer (NSCLC). For driver-negative NSCLC, ICIs are now the cornerstone of first-line therapy^[7]^. However, whether ICIs alone or in combination with other therapies would bring benefit to those with driver mutations is still to be elucidated. Gainor *et al*.^[8] ^reported poor response of ICI monotherapy in patients with epidermal growth factor receptor (EGFR) mutations or anaplastic lymphoma kinase (ALK) rearrangements. In the phase II ATLANTIC study, durvalumab showed activity in driver-positive NSCLC according to final OS analysis^[9]^. Therefore, continued research is required to explore the optimal use of ICI therapy in patients with driver mutations to improve outcomes of this cohort.

In our study, we retrospectively analyzed patients with locally advanced or metastatic non-squamous NSCLC that were treated with anti-PD-1 based mono- or combined-immunotherapy in Shanghai Pulmonary Hospital to assess the efficacy of ICIs on patients with driver-positive NSCLC.

## METHODS

### Study population

**Figure 1:**
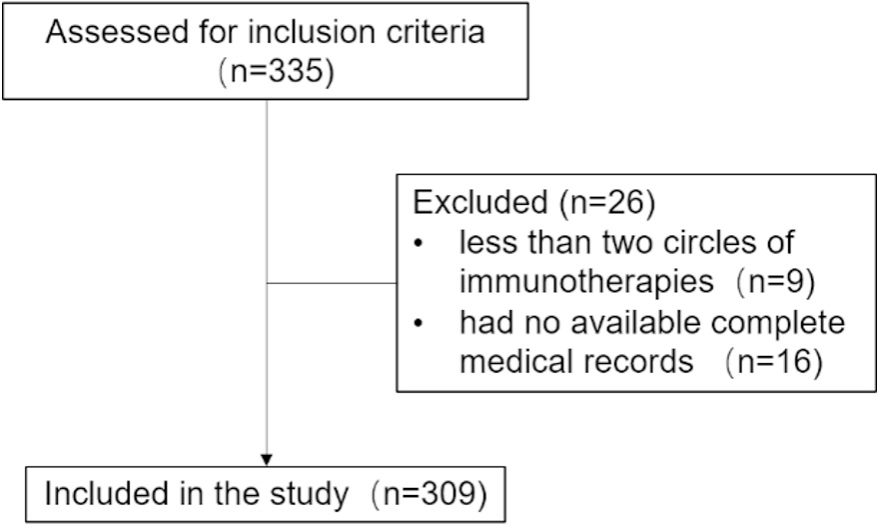
Flow chart of patient screening.

Patients with locally advanced or metastatic non-squamous non-small cell lung cancer from July 2015 to July 2020 in Shanghai Pulmonary Hospital were retrospectively enrolled. Inclusion criteria included the following: (1) a pathologic diagnosis of non-squamous non-small cell lung cancer; (2) testing data (either direct sequencing or NGS on validated platforms) for EGFR, V-Ki-ras2 Kirsten rat sarcoma viral oncogene homolog (KRAS), ALK, human epidermal growth factor receptor 2 (HER2), V-raf murine sarcoma viral oncogene homolog (BRAF), rearranged during transfection (RET), and c-ros oncogene 1 (ROS1); and (3) anti-PD-1-based monotherapy or combined immunotherapy as first-line or posterior-line therapy. Patients treated with fewer than two circles of immunotherapies and had no available complete medical records were excluded. The patient screening process is shown in Figure 1. We reviewed the medical records and abstracted the following patient characteristics: age, gender, Eastern Cooperative Oncology Group Performance Status (ECOG-PS), smoking history, histological type, clinical stage, mutation type, PD-L1 expression, details of treatment, and survival.

### PD-L1 analysis

PD-L1 immunohistochemistry was performed in Department of Pathology, Shanghai Pulmonary Hospital according to routine procedure. The antibody used was PD-L1 22C3 pharmDx (Dako, Carpinteria, CA, USA). PD-L1 positivity was defined as tumor proportion score cutoff of 1%.

### Statistical analysis

Patient characteristics are expressed as median and range for continuous variables and as frequencies and percentages for categorical variables. Progression-free survival (PFS) was measured from the date of first administration of PD-1 inhibitor treatment to disease progression or death due to any cause or last follow-up. Response Evaluation Criteria in Solid Tumors guidelines 1.1 (RECIST 1.1) was used to assess tumor response. The difference of response rate among different groups was calculated using Kruskal-Wallis test. Survival data were estimated using the Kaplan-Meier method and compared using the log-rank test in the overall cohort and subgroups. The data were analyzed using GraphPad Prism 8.0 and SPSS Statistics 20. A *P* value < 0.05 was considered statistically significant.

## RESULTS

### Patient characteristics

In this study, 309 non-squamous non-small cell lung cancer patients treated with anti-PD-1 based monotherapy or combined immunotherapy in Shanghai Pulmonary Hospital were identified. The median age was 61 years (range 20-88 years), 70.9% were male (219/309), and 55.7% (112/309) were never smokers. The majority of the patients had adenocarcinoma histology (98.4%, 304/309) and ECOG-PS of 0-1 at the start of immunotherapy (83.5%, 258/309). Detailed characteristics are shown in Table 1. In total, 182 received anti-PD-1 combined with chemotherapy (pemetrexed combined with carboplatin 85.7%, 156/182; paclitaxel-based chemotherapy 14.3%, 26/182), 42 received anti-PD-1 combined with antiangiogenic therapy (apatinib 90.5%, 38/42; bevacizumab 9.5%, 4/42). None of the enrolled patients received a combination of ICIs, chemotherapy, and antiangiogenic therapy. Among the whole cohort, 134 had genetic alteration, which involved EGFR (*n* = 81), KRAS (*n* = 31), ALK (*n* = 1), HER2 (*n* = 12), BRAF (*n* = 2), RET (*n* = 4), and ROS1 (*n* = 3). Among the 81 EGFR mutation patients, 42 had exon 19 deletions, 29 had exon20 L858R, and 10 had other mutations; 14 were harboring T790M mutation at the same time and 67 were not. The details of each subgroup are shown in Table 1.

**Table 1:** Characteristics of patients according to molecular alterations

	**All cases**	**EGFR**			**KRAS**		**ALK**	**HER2**	**BRAF**	**RET**	**ROS1**
		**19del**	**20 L858R**	**Other**	**G12C**	**G12D**	**EML4-ALK**	**20ins**	**V600E**	**RET-KIF5B**	**CD74-ROS1**
	***n *** **= 309**	***n *** **= 42**	***n *** **= 29**	***n *** **= 10**	***n *** **= 30**	***n *** **= 1**	***n *** **= 1**	***n *** **= 12**	***n *** **= 2**	***n *** **= 4**	***n *** **= 3**
Age	61 (20-88)	61 (20-72)	60 (40-75)	57 (38-73)	61 (49-75)	71	62	59 (45-68)	49, 61	49 (26-61)	41 (41-64)
Gender											
Male	219 (70.9%)	24 (57.1%)	18 (62.1%)	7 (70%)	27 (90%)	1 (100%)	1 (100%)	7 (58.3%)	2 (100%)	2 (50%)	1 (33.3%)
Female	90 (29.1%)	18 (42.9%)	11 (37.9%)	3 (30%)	3 (10%)	0 (0%)	0 (0%)	5 (41.7%)	0 (0%)	2 (50%)	2 (66.7%)
ECOG performance status											
0-1	258 (83.5%)	36 (85.7%)	24 (82.8%)	7 (70%)	26 (86.7%)	1 (100%)	0 (0%)	11 (91.7%)	0 (0%)	3 (75%)	3 (100%)
≥ 2	51 (16.5%)	6 (14.3%)	5 (17.2%)	3 (30%)	4 (13.3%)	0 (0%)	1 (100%)	1 (8.3%)	2 (100%)	1 (25%)	0 (0%)
Smoking history											
Current or former	137 (44.3%)	27 (64.3%)	22 (75.9%)	7 (70%)	11 (36.7%)	1 (100%)	0 (0%)	4 (33.3%)	1 (50%)	0 (0%)	2 (66.7%)
Never	172 (55.7%)	15 (35.7%)	7 (24.1%)	3 (30%)	19 (63.3%)	0 (0%)	1 (100%)	8 (66.7%)	1 (50%)	4 (100%)	1 (33.3%)
Histological type											
Adenocarcinoma	304 (98.4%)	41 (97.6%)	29 (100%)	10 (100%)	30 (100%)	1 (100%)	1 (100%)	11 (91.7%)	2 (100%)	4 (100%)	3 (100%)
NOS	5 (0.6%)	1 (2.4%)	0 (0%)	0 (0%)	0 (0%)	0 (0%)	0 (0%)	1 (8.3%)	0 (0%)	0 (0%)	0 (0%)
Clinical Stage											
Stage IIIB	2 (0.7%)	0 (0%)	1 (3.4%)	0 (0%)	0 (0%)	0 (0%)	0 (0%)	0 (0%)	0 (0%)	0 (0%)	0 (0%)
Stage IIIC	5 (1.6%)	1 (2.4%)	1 (3.4%)	0 (0%)	0 (0%)	0 (0%)	0 (0%)	0 (0%)	0 (0%)	0 (0%)	0 (0%)
Stage IV	302 (97.7%)	41 (97.6%)	27 (93.1%)	10 (100%)	30 (100%)	1 (100%)	1 (100%)	12 (100%)	2 (100%)	4 (100%)	3 (100%)
PD-L1 expression											
< 1%	72 (23.3%)	11 (26.2%)	5 (17.2%)	2 (20%)	5 (16.7%)	0 (0%)	0 (0%)	5 (41.7%)	0 (0%)	3 (75%)	2 (66.7%)
1%-49%	41 (13.3%)	2 (4.8%)	2 (6.9%)	0 (0%)	5 (16.7%)	0 (0%)	0 (0%)	2 (16.7%)	0 (0%)	0 (0%)	1 (33.3%)
≥ 50%	19 (6.1%)	3 (7.1%)	1 (3.4%)	0 (0%)	5 (16.7%)	0 (0%)	0 (0%)	0 (0%)	0 (0%)	1 (25%)	0 (0%)
Unknown	177 (57.3%)	26 (61.9%)	21 (72.4%)	8 (80%)	15 (50%)	1 (100%)	1 (100%)	5 (41.7%)	2 (100%)	0 (0%)	0 (0%)
Line of ICIs											
1	106 (34.3%)	1 (2.4%)	1 (3.4%)	4 (40%)	15 (50%)	0 (0%)	0 (0%)	6 (50%)	0 (0%)	2 (50%)	3 (100%)
2	127 (41.1%)	26 (21.9%)	17 (58.6%)	2 (20%)	12 (40%)	1 (100%)	0 (0%)	3 (25%)	0 (0%)	2 (50%)	0 (0%)
≥ 3	76 (24.6%)	15 (35.7%)	11 (37.9%)	4 (40%)	3 (10%)	0 (0%)	1 (100%)	3 (25%)	2 (100%)	0 (0%)	0 (0%)
Treatment modality											
Anti-PD-1 monotherapy	85 (27.5%)	2 (4.8%)	5 (17.2%)	1 (10%)	9 (30%)	1 (100%)	1 (100%)	4 (33.3%)	2 (100%)	1 (25%)	0 (0%)
Anti-PD-1 plus chemotherapy	182 (58.9%)	39 (92.9%)	22 (75.9%)	7 (70%)	17 (56.7%)	0 (0%)	0 (0%)	7 (58.3%)	0 (0%)	3 (75%)	3 (100%)
Anti-PD-1 plus antiangiogenic therapy	42 (13.6%)	1 (2.4%)	2 (6.9%)	2 (20%)	4 (13.3%)	0 (0%)	0 (0%)	1 (8.3%)	0 (0%)	0 (0%)	0 (0%)

ICIs: Immune checkpoint inhibitors; NOS: not otherwise specified; EGFR: epidermal growth factor receptor; KRAS: V-Ki-ras2 Kirsten rat sarcoma viral oncogene homolog; ALK: anaplastic lymphoma kinase; HER2: human epidermal growth factor receptor 2; BRAF: V-raf murine sarcoma viral oncogene homolog; RET: rearranged during transfection; ROS1: c-ros oncogene 1; ECOG: Eastern Cooperative Oncology Group.

### PD-L1 expression

**Figure 2:**
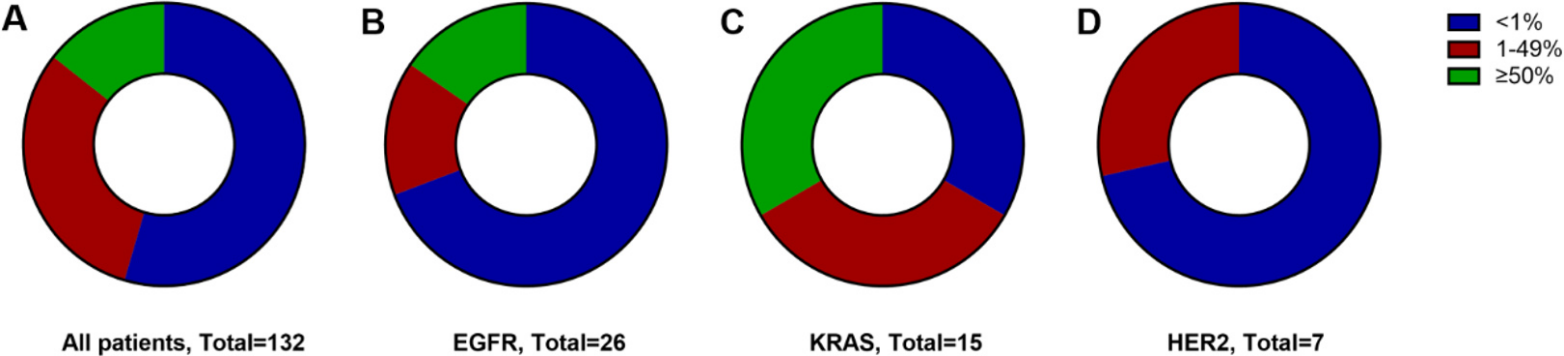
Categorical distribution of tumor PD-L1 expression: the whole cohort (A); EGFR-mutant NSCLC tumors (B); KRAS-mutant NSCLC tumors (C); and HER2-mutant NSCLC tumors (D). EGFR: Epidermal growth factor receptor; NSCLC: non-small-cell lung cancer; KRAS: V-Ki-ras2 Kirsten rat sarcoma viral oncogene homolog; HER2: human epidermal growth factor receptor 2.

PD-L1 expression status was available for 132 patients, of whom 54.5% (72/132) had less than 1% PD-L1 expression, 31.1% (41/72) had PD-L1 expression of 1%-49%, and 14.4% (19/132) had PD-L1 expression more than 50% [Figure 2A]. Looking into each subgroup, patients with EGFR mutation seemed to have a low PD-L1 expression (26 patients: 69.2%, 18/26 with PD-L1 < 1%, 15.4%, 4/26 with PD-L1 1%-49%, and 15.4%, 4/26 with PD-L1 ≥ 50%) [Figure 2B]. In the KRAS subgroup, the percentages of PD-L1 < 1%, 1%-49%, and ≥ 50% were all 33.3% (5/15) [Figure 2C]. Of the seven HER2 cases, 71.4% (5/7) had < 1% PD-L1 expression, while 28.6% (2/7) had a PD-L1 expression of 1%-49% [Figure 2D].

### Response rate

**Figure 3:**
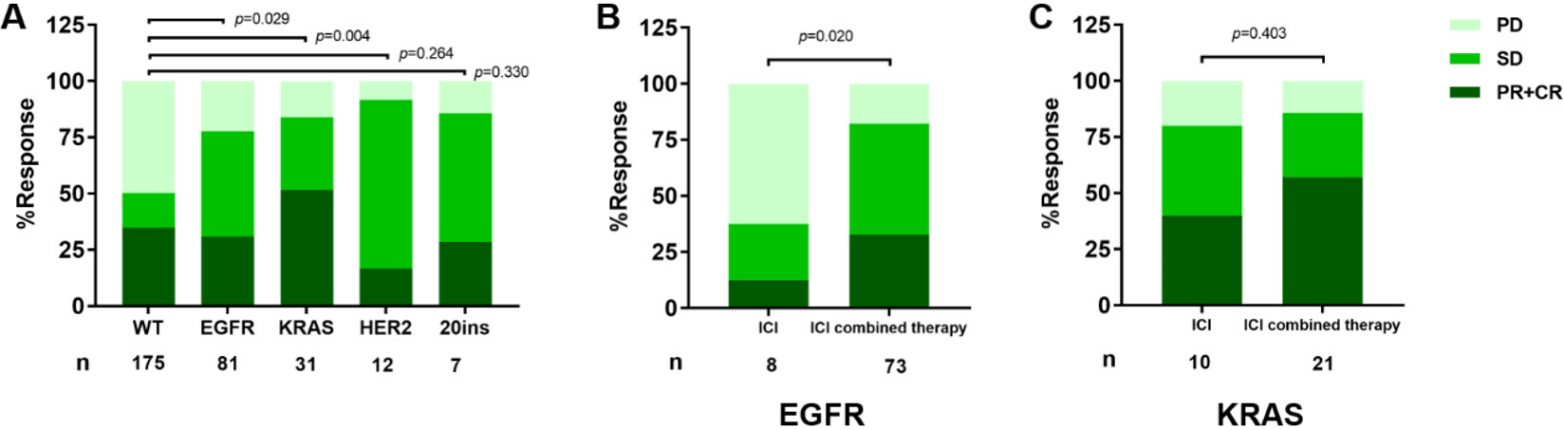
Response to ICIs according to Response Evaluation Criteria in Solid Tumors guidelines 1.1 (RECIST 1.1): (A) response to ICIs across molecular alterations; (B) response rate by treatment modality in EGFR patients; and (C) response rate by treatment modality in EGFR patients. ICIs: Immune checkpoint blockades; PD: progressive disease; SD: stable disease; PR: partial response; CR: complete response; WT: wild type. EGFR: epidermal growth factor receptor; KRAS: V-Ki-ras2 Kirsten rat sarcoma viral oncogene homolog.

Based on RECIST 1.1, the ORR of the patients with wild type (WT) was 34.9% and DOR was 50.3% (*n* = 175), while, in the EGFR subset, the ORR was 30.9% and DOR was 77.8% (*n* = 81). For KRAS patients, ORR was 51.6% and DOR was 83.9% (*n* = 31). The response rates in both the EGFR group and the KRAS group were statistically different from the WT group (*P* = 0.029 and *P* = 0.004, respectively) [Figure 3A]. In EGFR patients, the ICI combination therapy subset seemed to have better response rate compared to those who received ICI monotherapy (*P* = 0.020) [Figure 3B]. However, the difference was not found in KRAS patients [Figure 3C]. In 12 HER2 patients, ORR was 16.7% and DCR was 91.7%. While ORR was 28.6% and DCR was 85.7% in EGFR Exon20 insertion patients [Figure 3A].

### Progression-free survival

#### EGFR

**Figure 4:**
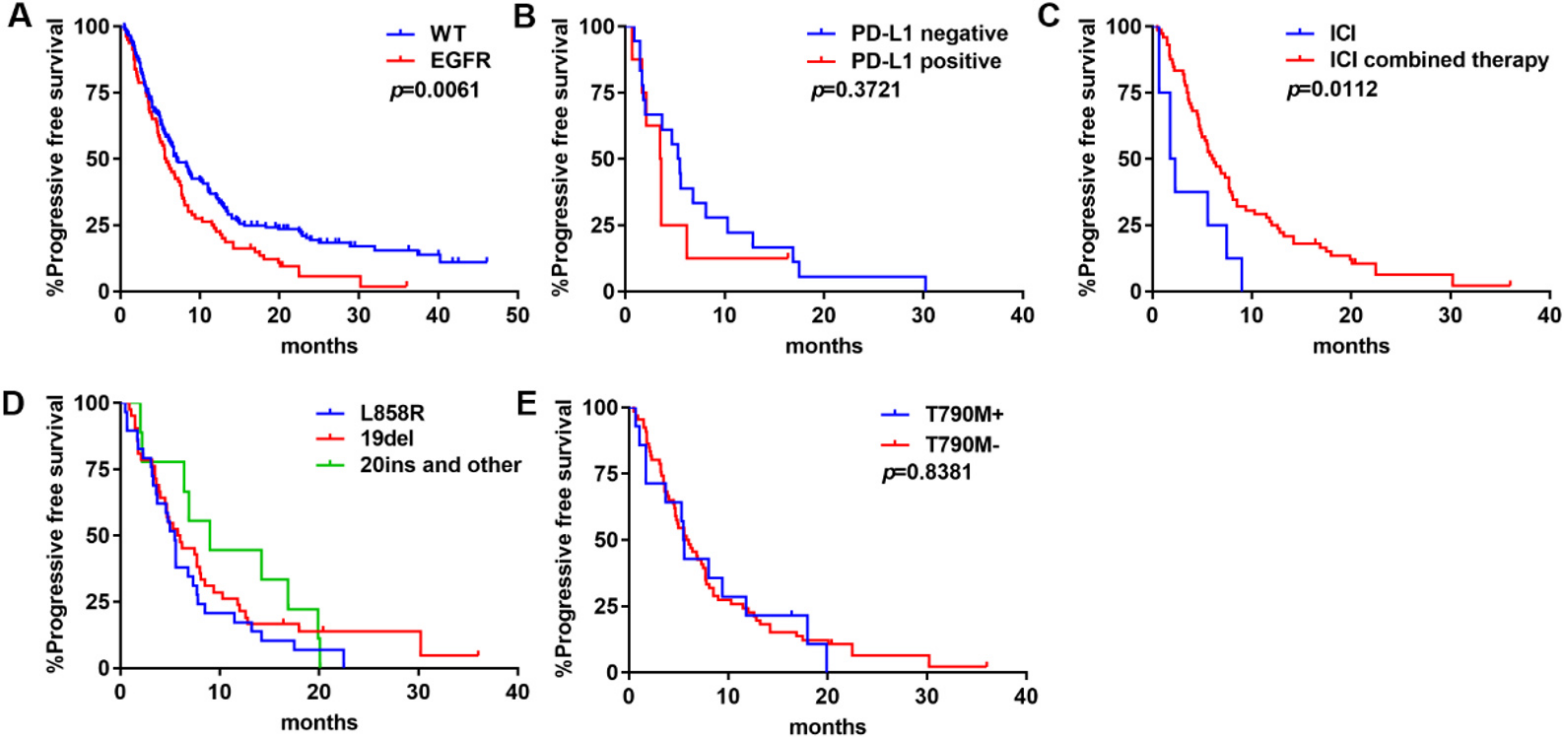
PFS of ICIs in EGFR-mutant NSCLC: (A) PFS in tumors with WT NSCLC or EGFR-mutant NSCLC; (B) PFS by PD-L1 expression levels; (C) PFS by treatment modality; (D) PFS by mutation type; and (E) PFS by T790M mutation status. ICIs: Immune checkpoint blockades; PFS: progression-free survival; WT: wild type; EGFR: epidermal growth factor receptor; NSCLC: non-small-cell lung cancer.

We investigated the outcomes of ICIs on patients with EGFR mutations. PFS of the EGFR subset was significantly shorter than that of the WT group (median PFS: 5.7 months *vs. *7.1 months; *P* = 0.0061) [Figure 4A]. Regarding PD-L1 expression, PFS was not significantly different (*P* = 0.3721) [Figure 4B]. ICI combined therapy was significantly correlated with a longer PFS compared with ICI monotherapy (median PFS: 7.7 months *vs. *4.7 months; *P* = 0.0112) [Figure 4C]. PFS was 5.5 months in L858R, 5.9 months in 19del, and 9.0 months in Exon20 insertion and other mutations, but the difference was not statistically significant among the three groups (*P* = 0.3411) [Figure 4D]. There was no difference in PFS between patients with or without T790M mutation (median PFS: 5.6 months *vs. *5.9 months; *P* = 0.8381) [Figure 4E].

#### KRAS

**Figure 5:**
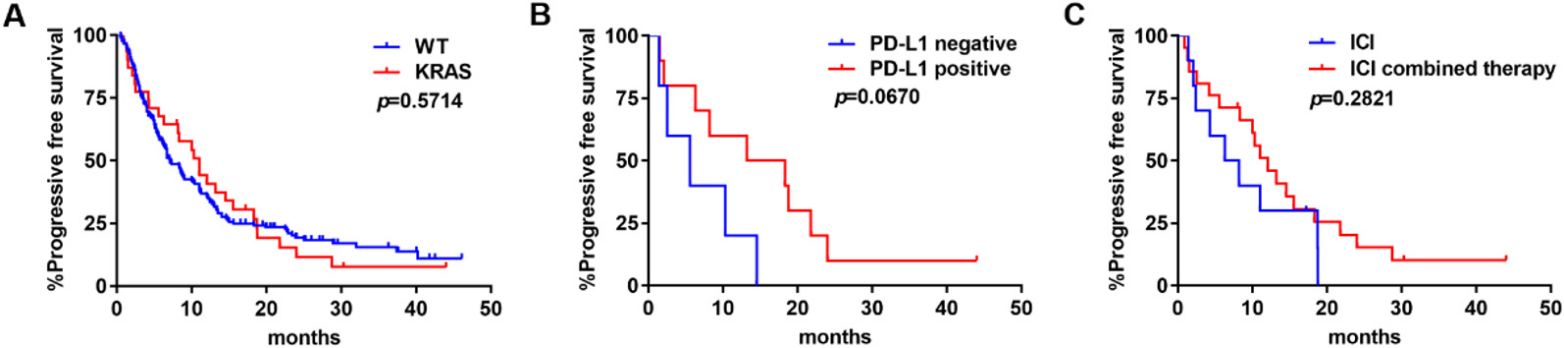
PFS of ICIs in KRAS-mutant NSCLC: (A) PFS in tumors with WT NSCLC or KRAS-mutant NSCLC; (B) PFS by PD-L1 expression levels; and (C) PFS by treatment modality. ICIs: Immune checkpoint blockades; PFS: progression-free survival; WT: wild type; KRAS: V-Ki-ras2 Kirsten rat sarcoma viral oncogene homolog; NSCLC: non-small-cell lung cancer.

KRAS was not associated with a benefit on PFS compared to those harboring no gene alteration (median PFS: 11.0 months *vs. *7.1 months; *P* = 0.5714) [Figure 5A]. In subgroup analyses, PD-L1 positive patients seemed to have longer PFS than PD-L1 negative ones, but the difference was not statistically significant (median PFS: 15.8 months *vs. *5.6 months; *P* = 0.0670) [Figure 5B]. ICI combined therapy had no advantage in PFS compared with ICI monotherapy (median PFS: 12 months *vs. *7.25 months; *P* = 0.5714) [Figure 5C].

#### Other molecular alterations

**Figure 6:**
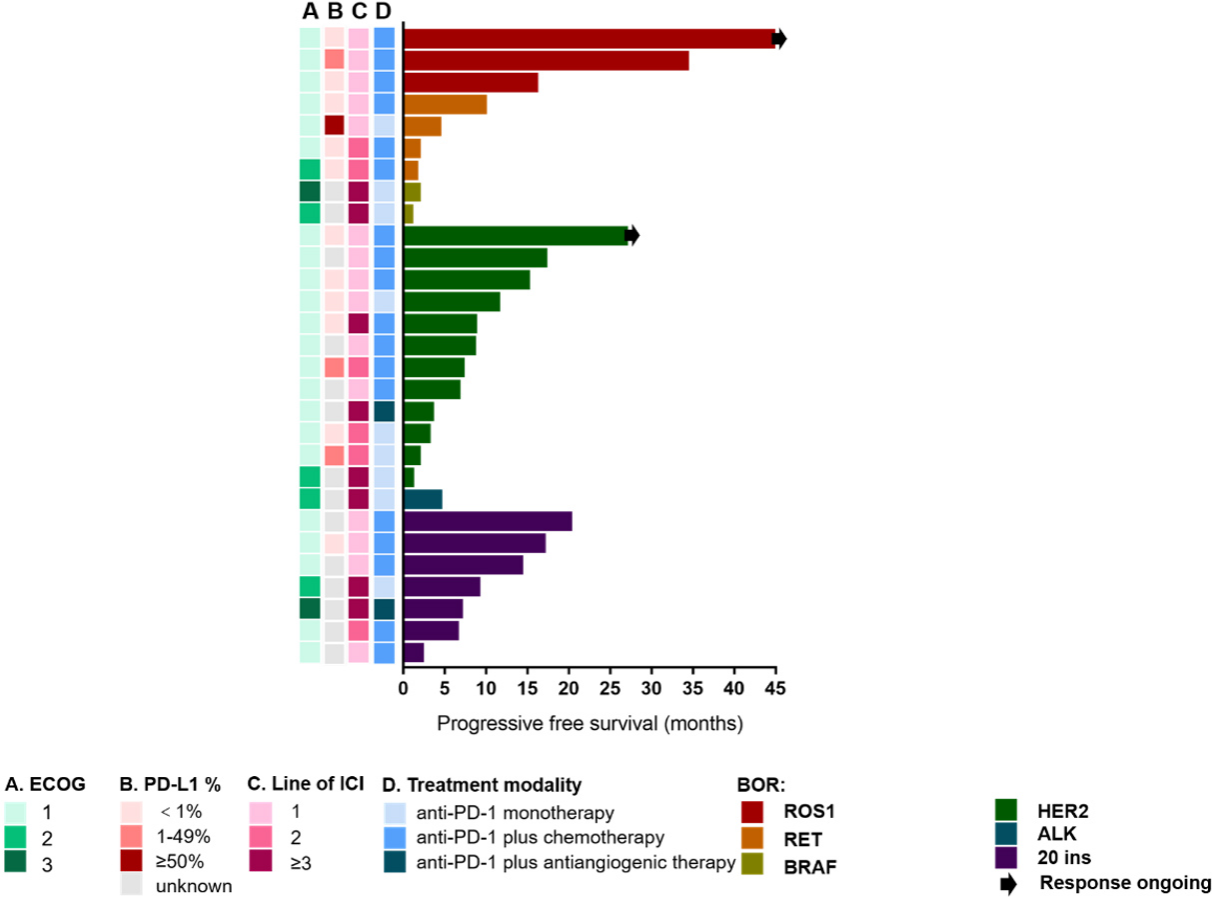
Swimmer’s plot of PFS across molecular alterations. PFS: Progression-free survival; ALK: anaplastic lymphoma kinase; HER2: human epidermal growth factor receptor 2; BRAF: V-raf murine sarcoma viral oncogene homolog; RET: rearranged during transfection; ROS1: c-ros oncogene 1; ECOG: Eastern Cooperative Oncology Group; ICI: immune checkpoint blockade.

All three patients with ROS1 rearrangements and ECOG 1 who received ICIs combined with chemotherapy as first-line treatment had response of PR. The PFS was 16.0, 34.2, and 45.0 months, respectively. The response of the patient with the longest PFS (45 months) was ongoing [Figure 6]. Four patients with RET rearrangements had PFS of 1.5, 1.8, 4.3, and 9.8 months. Three of them had ECOG 1 and one had ECOG 2. They received first-line (*n* = 2) and second-line (*n* = 2) ICI monotherapy (*n* = 1) or ICIs combined with chemotherapy (*n* = 3). No response was found in two BRAF patients, who received after second-line ICI monotherapy. Most HER2 patients had ECOG 1 (*n* = 11). Seven of them received ICIs combined with chemotherapy, four received ICI monotherapy, and one received ICIs combined with antiangiogenic therapy. ICIs as first-line therapy were observed in six patients, as second-line in three patients, and after second-line in three patients. The median PFS was 7.8 months (range 1.0-26.9 months). ALK rearrangements was identified in only one patient, whose PFS was 4.4 months, with ECOG 3, who received ICI monotherapy as after second-line treatment. We also enrolled seven patients with EGFR Exon20 insertion. The median PFS was nine months (range 2.2-20.1 months). They received ICIs combined with chemotherapy (*n* = 5) or antiangiogenic therapy (*n* = 1) or ICI monotherapy (*n* = 1), as first-line (*n* = 4), second-line (*n* = 1), or after second-line treatment (*n* = 2), with ECOG 1 (*n* = 5), 2 (*n* = 1), or 3 (*n* = 1).

## DISCUSSION

Targeted therapies against oncogene-driven NSCLC, including EGFR^[2]^, BRAF^[10]^, and HER2 mutations^[11]^ or ALK^[3]^, ROS1^[5]^, and RET^[12]^ rearrangements, have been proved to improve outcomes, and they form the standard first-line treatment in patients with advanced disease. However, progression inevitably occurs, and chemotherapy is currently the main subsequent treatment after TKI resistance. Considering the remarkable long-term benefits of ICI treatments, scientists have made lots of efforts to integrate ICIs into the treatment course of patients with oncogene-driven NSCLC. Mazieres *et al.*^[13]^ explored the activity of ICIs across NSCLC harboring oncogenic alterations and found driver-positive NSCLC exhibited poor response to ICI monotherapy. Hastings *et al.*^[14]^ reported more favorable outcomes of exon 21 mutations compared with exon 19 deletions in NSCLC treated with immunotherapy. Until now, the results on this topic are controversial.

In EGFR-driven NSCLC, initial clinical results indicate that ICIs have no clinical benefits. Meta-analysis^[15,16]^ of EGFR-mutated patients in Keynote 010, CheckMate 057, OAK, and POPLAR studies showed ICIs have poorer outcomes in cohorts with EGFR mutations compared to chemotherapy. We found similar findings in our research that patients with EGFR mutations had poor response to ICIs. This may be explained by the immunosuppressive and uninflamed tumor microenvironment (TME) and low tumor mutational burden (TMB), therefore being less immunogenic in the context of oncogenic addiction^[17,18]^. Besides the effect of oncogene mutation on TME, studies have shown that EGFR TKIs, chemotherapy, and antiangiogenic therapy also influence the TME. EGFR TKIs could increase infiltration of CD8+ T cells, dendritic cells, and M1 TAMs and inhibit regulatory T cells infiltration^[19]^. Bevacizumab, an antiangiogenic therapy, has been observed in melanoma to increase circulating CD8+ T cells and interleukin-6 levels so as to change the TME in combination with chemotherapy^[20]^. Therefore, the use of ICI-based combined therapy as post-line treatment after TKI resistance may be more effective. In the IMpower150 study, we did see prolonged PFS and OS in the ABCP (atezolizumab, bevacizumab, carboplatin, and paclitaxel) group compared with the BCP (bevacizumab, carboplatin, and paclitaxel) group in patients with EGFR-driven NSCLC^[21]^. In our study, we also found ICI combined therapy was correlated with better outcome than ICI monotherapy in EGFR-driven NSCLC patients.

In our study, we did not find any correlation between KRAS mutation and better survival, which is inconsistent with previous studies. This may be explained by the heterogeneity of KRAS-mutant NSCLC. KRAS mutation subtypes include G12C, G12D, G12V, G12A, and G13D, among others, and different KRAS mutations can activate distinct signaling pathways, leading to different downstream effects, which may result in different response to therapies^[22]^. Besides, KRAS mutation is always accompanied by different patterns of co-occurring mutations, which display different immune profiles and show varying sensitivities to ICIs. In KRAS-TP53 co-mutated tumors, increased expression of PD-L1, higher TMB, and a remarkable clinical benefit of ICIs was observed^[23,24]^. Conversely, KRAS-STK11 has always been associated with poor clinical response to ICIs^[24,25]^. Further studies are needed to differentiate patients suitable for different treatment options.

HER2 mutations have been reported in approximately 2%-5% of lung adenocarcinomas^[26,27]^ and correlated with poor prognosis^[27]^. The efficacy of ICIs in HER2-mutant NSCLC is ambiguous. Guisier *et al*.^[28] ^reported 23 patients harboring HER2 mutation treated with ICI monotherapy who had a response rate of 27%, which is close to that observed in unselected patients with NSCLC. Mazieres *et al*.^[13]^ reported an ORR of 7% and a median PFS of 2.5 months in 29 patients with HER2 mutation NSCLC treated with ICI monotherapy. In our study, ICI-based therapy displayed certain curative effect with 16.67% ORR, 91.7% DCR, and PFS of 7.8 months (range 1-26.9 months). For other rare driver mutations, the efficacy of ICIs on patients with ROS1 rearrangements or EGFR Exon20 insertion was rarely reported. In our study, three patients with ROS1 rearrangements had response of PR and durable PFS of 16.0, 34.2, and 45.0 months. Seven patients with EGFR Exon20 insertion had PFS of 9 months (range 2.2-20.1 months), ORR was 28.6%, and DCR was 85.7%. Therefore, ICI-based therapy may provide choices for these patients. As to BRAF, RET, and ALK, which represent a small subgroup of NSCLC, because of the limited number of patients, we could not draw a conclusion.

ICI-based combined therapy can bring benefit to patients with EGFR-mutant NSCLC. ICIs, especially ICI-based combination therapy, should not be excluded for patients with ROS1 rearrangement, HER2 mutation, and EGFR Exon20 insertion NSCLC. To achieve maximum benefit for these patients, better predictive biomarkers to select patients and combination modes of therapies should be explored.

## DECLARATIONS

### Copyright

© The Author(s) 2022.
